# Development of a model to predict gait independence in individuals with very severe gait disorder due to subacute hemiparetic stroke

**DOI:** 10.20407/fmj.2025-026

**Published:** 2025-11-05

**Authors:** Ryo Makino, Satoshi Hirano, Daisuke Imoto, Hiroki Kawanai, Makoto Watanabe, Takuma Ishihara, Shigeru Sonoda, Yohei Otaka

**Affiliations:** 1 Department of Rehabilitation Medicine, School of Medicine, Fujita Health University, Toyoake, Aichi, Japan; 2 Department of Rehabilitation, Fujita Health University Hospital, Toyoake, Aichi, Japan; 3 Department of Rehabilitation, Fujita Health University Nanakuri Memorial Hospital, Tsu, Mie, Japan; 4 Innovative and Clinical Research Promotion Center, Gifu University Hospital, Gifu, Gifu, Japan

**Keywords:** Cerebrovascular disease, Gait, Rehabilitation, Stroke, Walking

## Abstract

**Objectives:**

We aimed to develop and validate a model to predict gait independence at discharge from inpatient rehabilitation in individuals with subacute hemiparetic stroke who have very severe gait disorder.

**Methods:**

Overall, 298 individuals with subacute hemiparetic stroke and completely dependent gait were selected in one hospital as the training cohort. Seventy-seven individuals were selected in another hospital as the validation cohort. The prediction model was developed using multivariable logistic regression analysis, with individual characteristics selected based on a p-value threshold (<0.10) in the training cohort. Sensitivity, specificity, and area under the curve of the receiver operating characteristic curve were calculated in the training cohort, and external validation was conducted using the validation cohort.

**Results:**

In total, 102 (34.2%) and 40 (52.0%) individuals in the training and validation cohorts achieved independent gait while hospitalized, respectively. The prediction model factors were age, days from onset to admission, stroke type, affected side, severity of paresis, unaffected side function, and cognitive function. The sensitivity, specificity, and area under the curve in the training cohort were 0.81, 0.80, and 0.88, respectively. Corresponding values in the validation cohort were 0.82, 0.70, and 0.83, respectively.

**Conclusions:**

A model combining age, days from onset to admission, stroke type, affected side, severity of paresis, unaffected side muscle strength, and cognitive function effectively predicted gait independence at discharge in individuals with very severe gait disorder due to subacute hemiparetic stroke.

## Introduction

Individuals with stroke often experience gait disorders due to stroke-related functional impairments,^1,2^ which limit activity and social participation. Furthermore, gait disorders are critical determinant of long-term outcomes for individuals with stroke.^2^ Therefore, improving gait disorders is a key goal of stroke rehabilitation.^1–3^

Providing effective rehabilitation requires predicting the prospects for recovery of each individual, setting appropriate treatment goals, creating effective treatment plans, and supporting discharge planning.^4^ Several reviews have reported various factors that predict improvements in gait ability in individuals with stroke, which include the following: age, severity and strength of the paralyzed leg, cognitive function, presence of hemispatial neglect, incontinence, sitting ability, balance, sensory impairment, basic movement skills, and independence in activities of daily living (ADL).^5,6^ Other studies have reported tools for predicting improvements in gait ability in individuals with stroke.^7–9^

When focusing on individuals with severe disabilities caused by stroke, they often present with multiple disabilities, require specialized interventions, and spend high medical resources and costs.^10,11^ These individuals may demonstrate limited functional improvement through rehabilitiation,^11,12^ and intensive inpatient rehabilitation programs are often not provided because of perceived low cost-effectiveness.^11^ Nevertheless, individuals with severe gait disorder due to stroke may achieve positive outcomes. A cohort study focusing on individuals with severe gait and balance disorders due to stroke showed that 41%–53% of individuals with severe impairments at one week after starting rehabilitation had changes above the minimal detectable changes in gait and balance outcomes, which increased to 68%–84% at discharge.^13^ These findings suggest that identifying individuals with severe disabilities who are likely to show marked improvement is important. However, the individual characteristics associated with positive outcomes in individuals with very severe gait disorders due to stroke remain unclear. Several tools for predicting improvements in gait ability in individuals with stroke have been reported,^7–9^ although not limited to those with severe impairments. Therefore, identifying tools to predict rehabilitation outcomes for individuals with stroke who have very severe gait disorders is critical; such tools ensure that individuals in need receive appropriate treatment opportunities and contribute to effective healthcare allocations.

In this study, we aimed to develop and validate a prediction model for improvements in gait ability in individuals with very severe gait disorder due to hemiparetic stroke.

## Methods

### Study design

This was a two-center, retrospective, cohort study. The local Institutional Review Board approved the study protocol (HM24-323). The study was conducted in accordance with the principles of the Declaration of Helsinki and reported according to the Strengthening Reporting of Observational Studies in Epidemiology (STROBE) reporting guidelines.^14^ Informed consent was obtained via an opt-out method; the requirement for individual consent forms was waived. All individuals who did not opt out were included in the study.

### Participants

Individuals who were admitted to and discharged from a convalescent rehabilitation ward in a hospital (hospital A) between April 2006 and September 2021 were included as training cohort. Inclusion criteria involved individuals: (1) with hemiparesis caused by supratentorial intracerebral hemorrhage or cerebral infarction without a history of stroke, (2) admitted within 90 days of onset, and (3) with completely dependent gait. Individuals who experienced a change in condition that required transfer from the rehabilitation ward were excluded.

As a validation cohort, individuals admitted to and discharged from a convalescent rehabilitation ward of another hospital (hospital B) between January 2018 and December 2021 were enrolled. Hospital A and B belong to the same university. Inclusion and exclusion criteria were same as those for the training cohort except that individuals with missing data were excluded.

### Rehabilitation

All individuals received physical and occupational therapy, and those deemed necessary by rehabilitation physician also received speech-language therapy. Rehabilitation was provided for up to 180 minutes per day, in accordance with the Japanese system of convalescent rehabilitation,^15^ and all individuals received a minimum of 60 min of physical therapy daily. The rehabilitation program included the following training based on the individual’s condition: range of motion exercises, muscle strengthening, basic movement training (such as standing, sitting, transferring, and walking), training for ADL (such as eating, dressing, transferring, and toileting), swallowing training, speech training, and cognitive training. The primary goal of these programs was to improve individuals’ ADL.

### Data collection

The following variables related to individuals with stroke achieving gait independence as shown in previous studies^5,6^ were extracted from medical records in each of the training and validation cohorts: age, sex, stroke type, affected side, days from onset to admission, gait independence at admission, Stroke Impairment Assessment Set (SIAS)^16^ (including motor function in lower extremity, sensory function in lower extremity, trunk function, unaffected side function, and visuospatial cognitive function), and subtotal scores of Functional Independence Measure (FIM) motor and cognitive items.^17,18^ Furthermore, the degree of gait independence at the time of admission and discharge were collected.

The SIAS primarily involves single-task assessment of various functions and rates the individual’s performance on a scale (0–5 or 0–3).^16^ The reliability and validity of the SIAS have been confirmed in individuals with stroke.^19^ The motor function in lower extremity, sensory function in lower extremity, trunk function, and unaffected side function were summed to calculate the subtotal scores. Each of the three motor function items in lower extremity was scored on an ordinal scale from 0 to 5, resulting in a subtotal score ranging from 0 to 15. Sensory function in lower extremity, trunk function, and unaffected side function were each assessed using two items scored on an ordinal scale from 0 to 3, with a combined subtotal score ranging from 0 to 6. Visuospatial function was scored on an ordinal scale from 0 to 3. The SIAS was administered by the therapist at admission and recorded.

The FIM is one of the rating scales used for assessing independence in ADL.^17,18^ It consists of 13 motor items and 5 cognitive items. Each item is scored on an ordinal scale from 1 (complete dependence) to 7 (complete independence). The subtotal score of motor items ranges from 13 to 91, and that of cognitive items from 5 to 35. The reliability and validity of the FIM have been confirmed for individuals with stroke.^20^ The FIM was assessed and recorded by physicians, therapists, and nurses familiar with the scoring of the FIM. The subtotal scores of FIM motor and cognitive items were summed using each score of FIM motor and cognitive items.

A physical therapist scored gait independence during training based on the 7-point scale ranging from 1 to 7, in accordance with the concept of the proportion of assistance in the FIM walking item,^17,18^ same as a functional status score for the intensive care unit gait item.^21,22^ To assess pure gait ability, we selected gait independence during training as the evaluation measure.

### Statistical analysis

All statistical analyses were performed using R version 4.2.2 (2022-10-31) (The R Foundation for Statistical Computing, Vienna, Austria). A two-sided p-value <0.05 was considered statistically significant.

### Comparison of cohorts’ characteristics

Mean and standard deviation were calculated for continuous variables, medians and interquartile ranges for ordinal variables, and counts and percentages for categorical variables. To compare individuals’ characteristics between the training and validation cohort samples, the Mann–Whitney U test was used for ordinal and continuous variables, and the chi-squared test for categorical variables.

### Development of a prediction model

We analyzed the data following the procedure to develop the prediction model. We defined “independent” as gait independence score at discharge of 5 points or higher. Based on previous studies,^5,6^ we selected 11 explanatory variables related to gait independence, as follows: age, stroke type, affected side, days from onset to admission, subtotal score of SIAS motor function in lower extremity at admission, subtotal score of SIAS sensory function in lower extremity at admission, subtotal score of SIAS trunk function at admission, subtotal score of SIAS unaffected side function at admission, subtotal score of SIAS visuospatial cognition function, subtotal score of FIM motor item, and subtotal of FIM cognitive item. Missing data in the training cohort were imputed using multiple imputation method by chained equations (details for missing data are shown in the Supplementary table). The number of imputation iterations was set to 5. Multicollinearity was assessed using the variance inflation factor. The variance inflation factor was defined as ˂5, indicating no multicollinearity.^23^ To determine the explanatory variables to include in the prediction model, we conducted a multivariable logistic regression analysis using gait independence at discharge as the dependent variable and all variables without multicollinearity as the explanatory variables. The selection criteria for explanatory variables included in the prediction model were those with p<0.10 to identify potential predictors.^24^ A prediction model was developed using variables that met the criteria. Overfitting was assessed using optimism parameter. Optimism parameter assesses the magnitude of overfitting of model and is calculated using calibration plot and C-statistics. We determined that the model was not overfitted if the optimism is <0.2. Finally, we created a nomogram to visualize the relationship among the predicted probability and predictors. Nomograms are a way of graphically presenting statistical prediction models and quantify the probability of clinical events.^25^ By plotting the chart with the calculated diagram, the approximate calculation results are estimated.

### Validation of the prediction model

A multivariable receiver operating characteristic curves were plotted based on predicted values obtained from multivariable logistic regression model, and sensitivity, specificity, and area under the curve (AUC) values were calculated. Furthermore, to confirm the external validity of the developed prediction model, multivariable receiver operating characteristic curves were plotted using data collected from the validation cohort and developed prediction model, and sensitivity, specificity, cut-off values, and AUC values were calculated. The cut-off value was calculated based on the Youden Index. AUC values range from 0.5 to 1.0, with the following criteria: 0.5≤AUC<0.6 (fail), 0.6≤AUC<0.7 (poor), 0.7≤AUC<0.8 (fair), 0.8≤AUC<0.9 (good), and 0.9≤AUC (excellent).^26^

## Results

**Figure 1:**
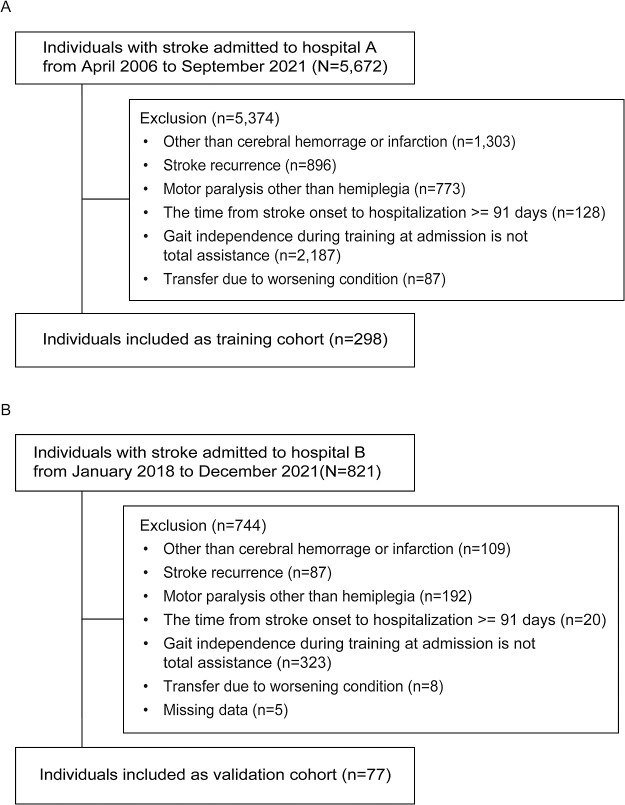
Flow chart of individuals in this study A. participants in the training cohort, B. participants in the validation cohort

Participants who met the inclusion and exclusion criteria were 298 and 77 in the training and validation cohorts, respectively (Figure 1). Among them, 102 (34.2%) and 40 (52.0%) individuals in the training and validation cohorts achieved gait independence at discharge, respectively. Table 1 shows the individuals’ characteristics in each cohort. Days from onset to admission was significantly higher in the training cohort than in the validation cohort. Furthermore, the score of motor function in lower extremity was significantly lower in the training cohort than in the validation cohort, although the score of sensory function in lower extremity, unaffected side function, visuospatial cognitive function, and subtotal of FIM motor item were significantly higher than in the validation cohort (Table 1).

**Figure 2:**
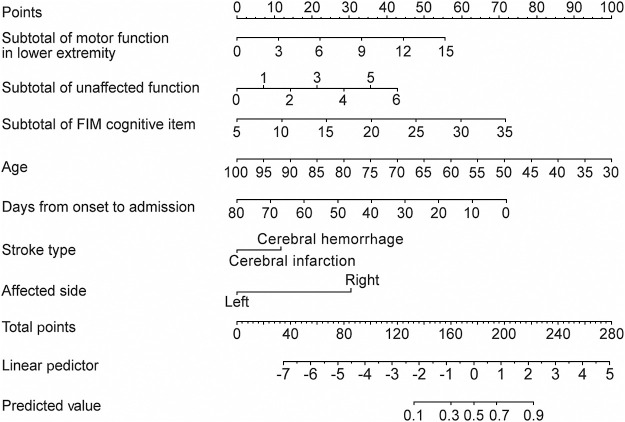
Nomogram for prediction gait independence at discharge in individuals with very severe gait disorder due to subacute hemiparetic stroke Draw a vertical line from the value of each variable to determine its corresponding point on the points scale. Sum these points to obtain the total score, which is then located on the total points scale. From this point, draw a vertical line downward to estimate the probabilities for the linear predictor and the predictor value.

No multicollinearity was found among the explanatory variables; hence, multivariable logistic regression analysis was performed using all explanatory variables. The multivariable logistic regression analysis identified age, days from onset to admission, stroke type, affected side, score of motor function in lower extremity, score of unaffected side function, and subtotal score of FIM cognitive item as significant explanatory factors (Table 2). Based on these findings, prediction models and nomograms were generated (Figure 2).

**Figure 3:**
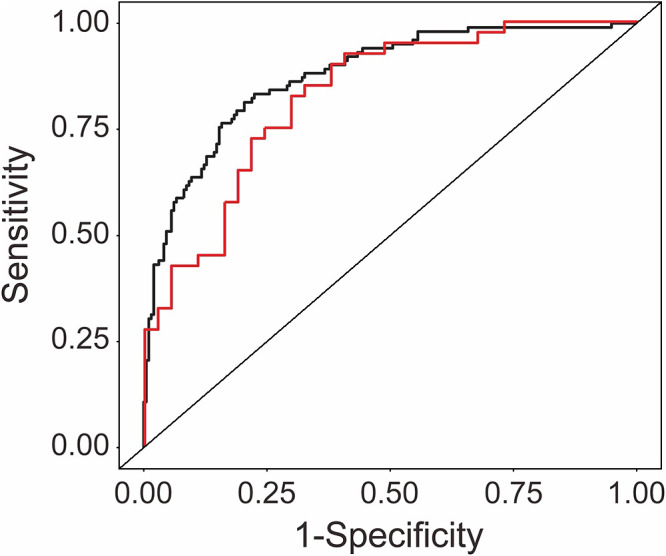
Multivariable receiver operating characteristics (ROC) in the training and validation cohorts The black line shows the multivariable ROC in the training cohort, and the red line shows the multivariable ROC in the validation cohort.

Using the training cohort data, multivariable receiver operating characteristic curve analysis demonstrated an AUC value of 0.88, indicating good accuracy, with a sensitivity of 0.81 and a specificity of 0.80 (Figure 3). External validation analysis using the validation cohort with the prediction model developed from the training cohort showed an AUC value of 0.83, with good accuracy, a sensitivity of 0.82, and a specificity of 0.70 (Figure 3).

## Discussion

This study developed a prediction model for gait independence at discharge in individuals with very severe gait disorder due to subacute hemiparetic stroke, admitted to a convalescent rehabilitation ward. The developed model included factors such as age, days from onset to admission, stroke type, affected side, severity of motor function in the affected lower extremity, unaffected side function, and cognitive function. Additionally, the prediction model demonstrated good accuracy in the training and validation cohorts, supporting its internal and external validity.

The prediction model developed in this study demonstrated good accuracy, achieving an AUC of 0.83 in external validation using a validation cohort. Kwah et al. developed a model combining stroke severity and age within 4 weeks post-stroke to predict gait independence at 6 months post-stroke, reporting an AUC of 0.84.^27^ Veerbeek et al. developed a model to predict gait independence at 6 months post-stroke using a combination of sitting balance and muscle strength on the affected side, assessed on days 2 and 9 after stroke onset.^7^ The model was externally validated in two cohorts, with reported AUCs of 0.68 and 0.80 for the prediction model based on day 2 from stroke onset, and 0.92 and 0.85 for the model based on day 9 from stroke onset.^28^ Although our model was developed using data from a single facility, it demonstrated external validity comparable to that of previous studies^27,28^ when evaluated in another facility with differing individual characteristics, including days from onset to admission and the severity of paresis. Maintaining predictive performance despite these variations suggests that the model might be generalizable to a broader range of clinical settings and individual populations.

To determine candidate variables for the prediction model, we evaluated those found to be associated with gait independence in previous studies.^5–9^ However, ADL, trunk function, visuospatial cognitive function, and lower extremity sensory function were not selected as explanatory variables. These variables were excluded from this prediction model for many reasons. First, ADL scores and trunk function may not have been selected because of the focus on severely disabled individuals. Regarding ADL score, a cohort study discovered that the Barthel Index (BI) predicts gait recovery in individuals with stroke. In that study, the group that regained gait had a mean BI of 50, whereas the group that did not regain gait had a mean BI of 3.^29^ This study assessed ADLs with the FIM, showing a median FIM motor subtotal of 17 (interquartile range: 13–22) in the training cohort. The conversion between FIM and BI has been reported,^30^ suggesting that the participants in this study are similar to those who did not regain gait in the previous study.^30^ Several studies have reported that trunk function is key in predicting gait recovery.^7,8,31,32^ Individuals in this study had lower trunk function than in the previous studies, although the evaluation methods differ.^7,8,31,32^ Therefore, the participants in this study had more severe impairments in ADL and trunk function, leading to these variables not being selected. Second, regarding visuospatial cognitive function, a systematic review revealed that the relationship between hemispatial neglect and gait independence is controversial.^33^ A study reported that gait recovery is unaffected by hemispatial neglect severity after adjusting for paresis.^34^ In contrast, a study reported a weak relationship between hemispatial neglect improvement and gait recovery, with paresis improvement having a stronger impact.^35^ Therefore, this study may have prioritized more influential factors, such as severity of motor function. Finally, regarding sensory impairment, gait recovery is more difficult in individuals with both motor paresis and sensory impairment than in those with motor paresis alone.^9,36^ However, a study reported that sensory impairment was not an independent predictor using a prediction model based on multiple regression analysis.^4^ The results of the multivariable logistic regression analysis in this study showed that sensory disorder was not selected as an explanatory variable in the model, which was consistent with the previous report.^4^

The clinical significance of this study is that the developed model for predicting gait independence in individuals with very severe gait disorder due to subacute hemiparetic stroke demonstrated high validity. Furthermore, the results suggest that the selected explanatory variables play a crucial role in gait recovery and provide valuable insights for future research. No reliable method has been available to predict rehabilitation outcomes in individuals with severe hemiparetic stroke, potentially leading to inadequate allocation of medical resources.^11^ If this model can help identify individuals who are likely to benefit from rehabilitation, it can enable more effective distribution of medical resources to those in need. Additionally, the model is highly practical for clinical use, as the included variables can be safely and easily assessed while the individual is seated in a wheelchair. Moreover, the nomogram visualizing the developed model could be used at the bedside by clinicians without the need for complex calculations or specialized computer software. Clinicians could predict the probability of achieving gait independence by scoring each variable and calculating the total score in each case. If the estimated probability is high, clinicians may place greater focus on gait training, whereas if it is low, they might focus more on practicing alternative mobility methods. These adjustments based on the prediction could help support clinical decisions, such as planning individualized rehabilitation programs.

### Study limitations

This study has several limitations. Facility bias may have been present, as samples from a single facility were used to develop the prediction model. In addition, although the training and validation cohorts were sampled from two different hospitals, these facilities are affiliated with the same university and may share some personnel. Therefore, they might not be considered completely independent, as treatment policies may be similar. A more accurate model could be obtained using samples from multiple facilities. Increasing the number of individuals in the training and validation cohorts may further enhance the accuracy and validity of the model.

## Conclusions

The developed model to predict gait independence at discharge in individuals with very severe gait disorder due to subacute hemiparetic stroke had high validity. The model will be useful for planning the rehabilitation program for individuals with very severe gait disorder due to subacute hemiparetic stroke.

## Figures and Tables

**Table 1:** Individuals’ characteristics in the training and validation cohorts

Variables	Training cohorts (n=298)	Validation cohorts (n=77)	p-value
Age, years, median (IQR)	73 (65–80)	75 (62.5–80)	0.839
Sex, men, number (%)	149 (50.0)	45 (58.4)	0.186
Stroke type, cerebral infarction, number (%)	141 (47.3)	30 (39.0)	0.190
Affected side, right, number (%)	133 (44.6)	33 (42.9)	0.780
Days from onset to admission, median (IQR)	34 (24–46)	22 (13–32.5)	<0.001
SIAS, points, median (IQR)			
Subtotal score of motor function in lower extremity	0 (0–0)	0 (0–5)	<0.001
Subtotal score of sensory function in lower extremity	2 (0–3)	0 (0–1)	<0.001
Subtotal score of trunk function	2(0–3)	2 (0–4)	0.680
Subtotal score of unaffected function	4 (2–5)	2 (1–3)	<0.001
Visuospatial function score	2 (1–3)	1 (0–3)	0.043
FIM, points, median (IQR)			
Subtotal score of motor items	17 (13–22)	14 (13–21)	0.010
Subtotal score of cognitive items	14 (8–20)	12 (7–20)	0.347

IQR, interquartile range; FIM, Functional Independence Measure, SIAS, Stroke Impairment Assessment Set

**Table 2:** Results of multivariable logistic regression analysis

Variables	Odds ratio (95% CI)	p-value
Subtotal score of motor function in lower extremity	1.206 (1.057–1.377)	0.006
Subtotal score of sensory function in lower extremity	0.985 (0.788–1.230)	0.890
Subtotal score of trunk function	1.025 (0.817–1.287)	0.827
Visuospatial function score	0.995 (0.712–1.391)	0.977
Subtotal score of unaffected function	1.315 (1.015–1.705)	0.038
Subtotal score of FIM motor items	1.030 (0.970–1.094)	0.327
Subtotal score of FIM cognitive items	1.110 (1.045–1.178)	0.001
Age	0.932 (0.903–0.962)	<0.001
Days from onset to admission	0.957 (0.934–0.980)	<0.001
Stroke type (cerebral infarction over cerebral hemorrhage)	1.828 (0.936–3.571)	0.077
Affected side (right over left)	0.244 (0.109 –0.543)	0.001

CI, confidence interval; SIAS, Stroke Impairment Assessment Set; FIM, Functional Independence Measure
