# Supplementary material for: Development of a model to predict gait independence in individuals with very severe gait disorder due to subacute hemiparetic stroke
Source: Fujita Med J. 2025 Nov 5;12(1):60–6. doi: 10.20407/fmj.2025-026 (PMC12865285; doi:10.20407/fmj.2025-026)
Supplement: Supplementary file 1 — Supplementary Table [file fmj-12-060-s001.pdf]

Supplementary table. Missing data in the training cohort data

Variables	n = 298
Visuospatial function score	22 (7.38)
Subtotal score of unaffected function	21 (7.05)
Subtotal score of sensory function in lower extremity	14 (4.70)
Subtotal score of motor function in lower extremity	9 (3.02)
Subtotal score of trunk function	3 (1.01)

Data shown are numbers (%).

SIAS, Stroke Impairment Assessment Set
